# Short-Term Outcome of Rehabilitation Program with Hybrid Assistive Limb after Tendon Lengthening in Patients with Cerebral Palsy

**DOI:** 10.3390/pediatric14040059

**Published:** 2022-11-12

**Authors:** Mayumi Matsuda Kuroda, Hirotaka Mutsuzaki, Shogo Nakagawa, Kenichi Yoshikawa, Kazushi Takahashi, Yuki Mataki, Ryoko Takeuchi, Nobuaki Iwasaki, Masashi Yamazaki

**Affiliations:** 1 Department of Physical Therapy, Ibaraki Prefectural University of Health Sciences, 4669-2 Ami, Ibaraki 300-0394, Japan; 2 Center for Medical Science, Ibaraki Prefectural University of Health Sciences, 4669-2 Ami, Ibaraki 300-0394, Japan; 3 Department of Orthopedic Surgery, Ibaraki Prefectural University of Health Sciences Hospital, 4733 Ami, Ibaraki 300-0331, Japan; 4 Department of Orthopedic Surgery, Tsukuba Park Family Clinic, 485-1, Tsukuba 300-2654, Japan; 5 Department of Physical Therapy, Ibaraki Prefectural University of Health Sciences Hospital, 4733 Ami, Ibaraki 300-0331, Japan; 6 Department of Rehabilitation Medicine, University of Tsukuba Hospital, 2-1-1 Tsukuba, Ibaraki 305-8576, Japan; 7 Department of Pediatrics, Ibaraki Prefectural University of Health Sciences Hospital, 4733 Ami, Ibaraki 300-0331, Japan; 8 Department of Orthopedic Surgery, Faculty of Medicine, University of Tsukuba, 1-1-1 Tsukuba, Ibaraki 305-8577, Japan

**Keywords:** soft tissue surgery, muscle lengthening, robotics, exoskeleton device, motor activity, physical functional performance, cerebral palsy

## Abstract

In this study, we aimed to evaluate the short-term outcomes of a rehabilitation program with the Hybrid Assistive Limb^®^ after soft tissue lengthening in young patients with cerebral palsy. We assessed six patients with cerebral palsy who underwent soft tissue surgery followed by gait training using the Hybrid Assistive Limb^®^. Clinical assessments were conducted preoperatively, before, immediately after, and at 1, 2, and 3 months after gait training. Gross Motor Function Measure was improved 5.93 ± 6.11% (mean ± standard deviation, *p* < 0.05), Canadian Occupational Performance Measure performance was improved 3.12 ± 1.53 points, and satisfaction was improved 3.80 ± 2.14 points (*p* < 0.05). The knee extension strength on the operated side was changed 7.75 ± 4.97 Nm after the intervention (*p* = 0.07). In ambulatory patients, gait speed was changed 8.37 ± 1.72 m/min, stride length was changed 10 ± 6.16 cm, and 6 min walking distance was changed 52 ± 16 m after the intervention. Training with the Hybrid Assistive Limb^®^ may improve walking ability and clinical outcomes in young patients with cerebral palsy after soft tissue lengthening.

## 1. Introduction

Cerebral palsy (CP) is the most common physical disability in childhood. Although CP is a non-progressive neurological lesion resulting in motor impairment, the condition is not unchanging, and various impairments of the neuromuscular and musculoskeletal systems become features of CP. The most common type of CP is hypertonia/spasticity. Crouch or equinus gait is the most common gait problem in patients with CP [1,2]. Orthopedic surgical therapies, including muscle lengthening, tenotomy, and/or osteotomy, have achieved good results [3]. However, after surgical intervention, a decline in walking ability due to a change in load balance, reverse deformation caused by muscle weakness during fixation, and joint contracture recurrence have been reported [4]. Therefore, we focused on early gait training using an exoskeleton robot suit to improve postoperative muscle weakness.

The Hybrid Assistive Limb^®^ (HAL; CYBERDYNE, Tsukuba, Japan) used in this study is a novel robotic device that can assist voluntary walking based on the wearer’s intention. HAL has been reported to be beneficial for gait training in patients with CP [5,6,7]. HAL assists the user in controlling hip and knee motion voluntarily, based on signals from force/pressure sensors in the shoes or even weak bioelectric skin surface signals [8]. HAL aids patients with severe CP move their hip and knee joints as desired, even facilitating gait training. Gait training with HAL is reportedly effective in patients with decreased walking ability following tendon lengthening procedures for crouch gait pattern [5].

The effect of HAL after soft tissue lengthening has been previously reported; however, the report was based on a single CP case [5]. By expanding the study design to a case series (level of evidence IV) with six similar individuals who underwent the same surgery-rehabilitation regime, this study evaluated the gait and gross motor function outcomes of HAL integration into a postoperative rehabilitation program for young individuals with CP who had multiple joint contractures and received multi-level lower limb soft tissue lengthening. We hypothesized that HAL training can be safely employed in the early postoperative period after soft tissue surgery in cases of muscle weakness, and HAL training may improve the range of motion, walking ability, and motor function in CP patients.

## 2. Materials and Methods

### 2.1. Patients

Between March 2017 and April 2019, we enrolled patients with CP who were scheduled to undergo tendon lengthening in their lower limbs. All tendon lengthening procedures were performed by three experienced pediatric orthopedic surgeons. The inclusion and exclusion criteria of the study were in accordance with those described in previous studies involving robot training [6]. Briefly, the inclusion criteria were as follows: (i) Gross Motor Function Classification System (GMFCS) levels I–IV, (ii) ability to participate in other conventional rehabilitation exercises during hospitalization, and (iii) ability to signal pain, fear, and discomfort reliably, using verbal or nonverbal signals. The exclusion criteria were as follows: (i) difficulty wearing the HAL due to severe joint deformation and/or contracture, (ii) lack of patient cooperation, (iii) inadequate fitting of the HAL device, and (iv) seizure disorders not fully controlled by medications. The study participants were six patients with CP (mean age, 16.0 range, 11–24 years; four male participants) who were scheduled to undergo tendon lengthening in their lower extremities (Table 1). All participants and/or their guardians provided informed consent for inclusion before participating in this study. The study was conducted according to the Declaration of Helsinki, and the protocol was approved by the Ethics Committee of Ibaraki Prefectural University of Health Sciences (Project identification codes, 682, e83, and e119).

### 2.2. HAL Intervention

**Figure 1:**
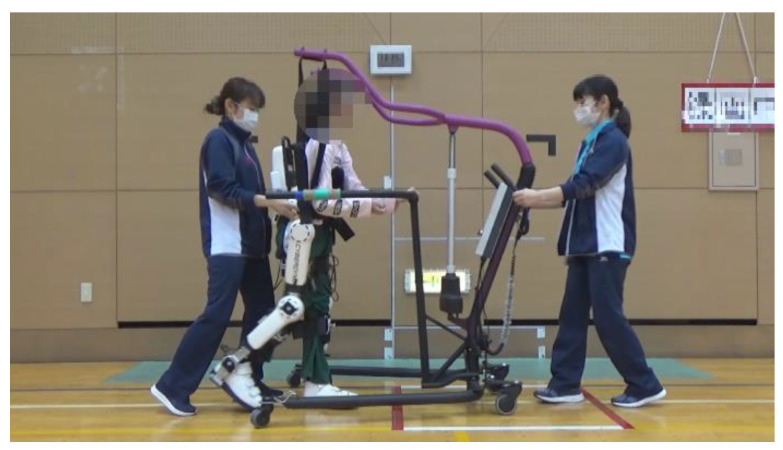
Hybrid Assistive Limb^®^ (HAL) device for medical use (lower limb type).

Training using HAL lower limb type S size was initiated after the postoperative fixation period and after the surgeon determined that standing training was possible. Participants underwent the training as reported by Matsuda et al. [6]: approximately 20 min per session, 2–4 times per week for 4 weeks, for a total of 12 sessions, along with usual physical therapy such as muscle stretching or strengthening. The gait training with HAL was performed during the in-patient period. The control mode at the time of intervention was the Cybernic Autonomous Control (CAC) and/or Cybernic Voluntary Control (CVC), depending on patient ability. Before training, flexion/extension balance and assist torque at the hip and knee joints were optimized according to the individual electromyogram signal. To avoid falling, a walking device (All-in-One Walking Trainer, Ropox A/S, Naestved, Denmark) with a harness was used (Figure 1).

While walking and wearing HAL, patients utilized a walking device (All-in-One Walking Trainer) with a harness for safety.

### 2.3. Assessments

During gait training using HAL, the presence of any adverse events, including surgical site infection, fracture, contracture deterioration, severe pain, and re-rupture were assessed on weekdays by three experienced pediatric orthopedic surgeons. We also reviewed the training details and analyzed the patient’s heart rate and degree of fatigue using a Borg scale. The range of motion of the knee or ankle joint that underwent tendon lengthening was measured at pre-surgery, pre-gait training using HAL, and post-gait training using HAL.

Outcome measures were previously defined [6]. The primary endpoint was Gross Motor Function Measure (GMFM), Pediatric Evaluation of Disability Inventory (PEDI), Canadian Occupational Performance Measure (COPM), and maximum isometric knee extension strength using a hand-held dynamometer (HHD, Mobile MT-100, SAKAI, Tokyo, Japan). GMFM allows quantitative evaluation of motor function. GMFM consists of 88 items that have been categorized into 5 dimensions of gross motor function: (1) lying and rolling; (2) sitting; (3) crawling and kneeling; (4) standing; and (5) walking, running, and jumping. Each item is scored using a 4-point Likert scale (0: does not initiate; 1: initiates; 2: partially completes; and 3: completes) [9]. PEDI is used for assessing a child’s capability and performance in daily life. In this study, we used the PEDI-Functional Skills Scale (PEDI-FSS) to measure the child’s actual performance in three domains: self-care, mobility and social functioning in daily environments. PEDI-FSS is measured by reports of whether the child is capable of performing each of 197 tasks in those three domains. All items were checked as either capable (score 1) or unable (score 0) [10]. COPM is a client-centered measure designed to detect a change in a person’s perception of their occupational performance in self-care abilities, productivity, and leisure activities [11,12]. COPM identifies deficits in occupational performance or problems with everyday activities that are most important to the patient, and they rate their perceptions of the importance of each activity using a 10-point scale. The five most important activities were further assessed using a 10-point scale on two scales: performance and satisfaction with performance. The performance and satisfaction scores of the selected activities are summed and averaged over the number of problems, and a total performance and satisfaction score out of 10 was generated, respectively. A two-point change in self-rated motor performance and satisfaction as assessed by COPM is considered clinically significant [13]. Maximum isometric knee extension muscle force was measured as isometric maximal voluntary muscle contraction over 5 s was performed three times on each side, and the maximum value on each side was used. The distance (m) from the center of the knee joint to the center of the sensor belt was measured, and the muscle force was converted to torque (N · m) for analysis [6,14].

As a secondary outcome measure, we examined gait speed (m/s), step length (cm), and cadence (step/min) during self-selected walking speed (SWS) and maximum walking speed (MWS) over the 10 m walking test (10MWT), and the 6 min walking distance (6MD), physiological cost index (PCI) during the 6 min walking test. Additionally, gait parameters, including gait speed, step length, cadence, 6MD, and PCI, were measured in patients who could walk without assistance from others. Some patients used a walker and ankle orthoses. Measurements were performed before surgery, before and after the intervention, and at 1, 2, and 3 months after intervention using HAL. Measurements before surgery and before and after the intervention were conducted during hospitalization within 5 days from the first and the last day of the intervention. Measurements at 1, 2, and 3 months after the intervention were performed in hospitalization or outpatient setting within 7 days before and after each reference date. The COPM was obtained before and after the intervention and at 1, 2, and 3 months after the intervention with HAL; it was not measured before surgery.

### 2.4. Statistical Analysis

Non-parametrical analyses were applied because of the small sample size. The differences were tested using Wilcoxon Signed Rank Test for baseline (before surgery and before intervention) and in change scores between baseline to after HAL intervention (immediately, 1, 2, 3 months), respectively. After HAL intervention (immediately, 1, 2, 3 months) were tested separately against Baseline in order to reduce the effect of missing data. Significance level was set at *p* < 0.05. All statistical analyses were performed using IBM SPSS Statistics, version 28.0 (IBM Japan, Tokyo, Japan). For the small number of cases, some data were limited to simple numeric observation; no formal statistical significance testing was performed.

## 3. Results

All patients underwent various soft tissue surgeries at our institution: combined medial and lateral hamstring lengthening for knee flexion contracture (two patients); Achilles’ tendon lengthening for equinus foot (two patients); and combined Achilles’ tendon and posterior tibial tendon lengthening for equinovarus foot (two patients).

All participants completed the protocol of 12 sessions gait training intervention with HAL without any adverse events. Training using the HAL was performed 2–4 times per week according to protocol. The average period of training was 23.5 (19–29) days (Table 1). Training using HAL lower limb type S size was initiated after the postoperative fixation period (28 [14–42] days on average) and after the surgeon determined that standing training was possible. The mean period of starting HAL training from surgery was 50 (36–64) days (Table 1). In the present patients, there was a large variation in the period from surgery to the starting day of HAL. The period from the date of surgery to the date of HAL training initiation ranged from 36 to 47 days in patients who underwent Achilles’ tendon lengthening, whereas it ranged from 62 to 64 days in patients who underwent hamstring lengthening. Patients who underwent hamstring lengthening took longer to be able to be standing training than those who underwent Achilles’ tendon lengthening (Table 1).

**Figure 2:**
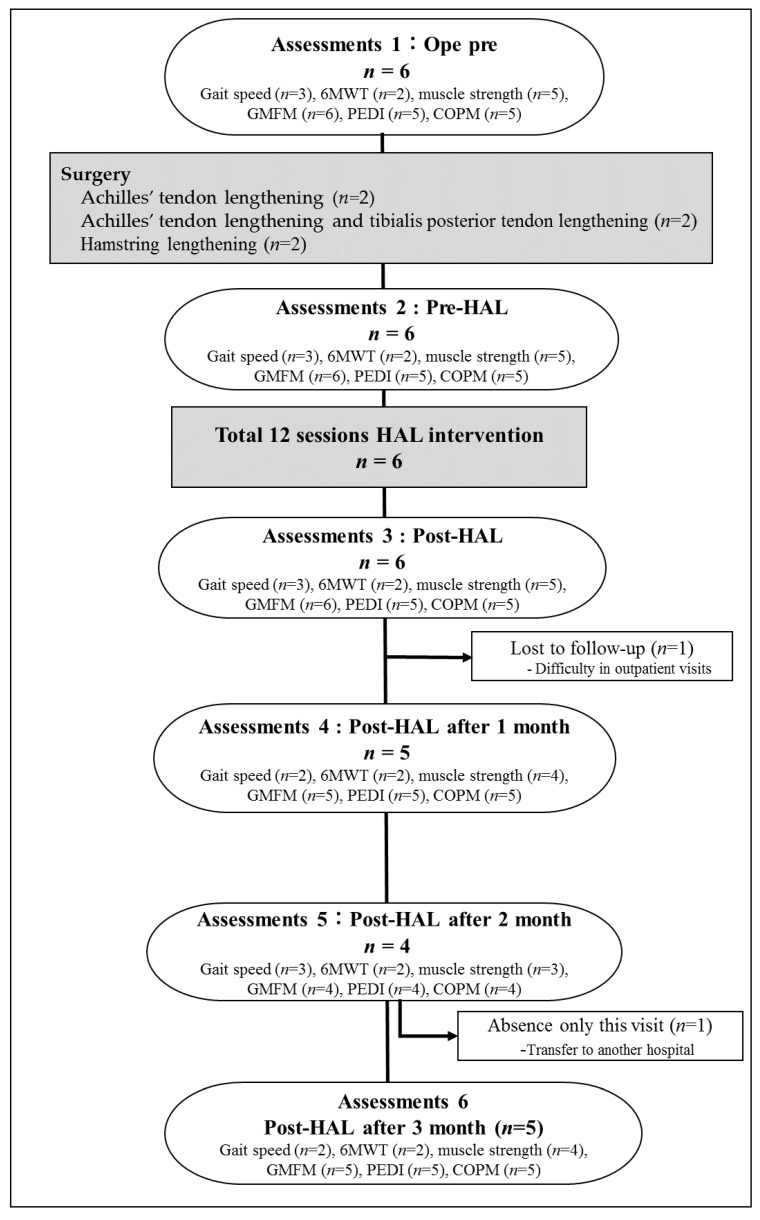
Exercises with the Hybrid Assistive Limb^®^ (HAL) device.

Patient 6 could not be evaluated since the patient moved to a different hospital 2 months after the intervention. In patient 3, a 10 m walking test on the MWS, 6MD, PCI, PEDI, and COPM was not measured, and follow-up evaluation was not conducted because the patient was having difficulty staying motivated and was unable to come to our hospital after discharge (Figure 2).

The total GMFM score in all patients increased after HAL intervention compared with that before HAL. GMFM showed improvement after 3 months of HAL intervention compared to before surgery (*p* = 0.043). In addition, improvement was observed after HAL (*p* = 0.027), 1 month after HAL (*p* = 0.043), and 3 months after HAL (*p* = 0.042) compared to before HAL intervention. The GMFM total scores in two patients who underwent hamstring lengthening were significantly decreased before the HAL intervention compared with preoperative values because they could not move (flex and extend) the knee joints due to postoperative pain. On the other hand, the hamstring lengthening patients had a significantly increased GMFM total scores after HAL, compared to those who underwent Achilles tendon lengthening. They continued to improve GMFM not only immediately after HAL intervention, but also up to 3 months post HAL. In patients 3 and 4, dimensions C (crawl and kneel) and D (stand) showed improvement after HAL intervention and were maintained for 3 months post-HAL. In patient 2, dimension E was improved by the operation, and the score was further increased by the HAL intervention for 3 months (Table 2).

The PEDI was unchanged in patients 2, 4, and 5 during the pre-/postoperative period and throughout the HAL intervention. Concurrently, in patient 1, a point was added to the evaluation item of outdoor movement and stairs going up and down 1-month post-HAL, compared with that seen preoperatively. In patient 6, the score was lowered due to difficulties in crawling or kneeling on the floor because of postoperative pain during flexion of the knee joint caused by knee extension fixation; however, it improved to the preoperative value 3 months after the HAL intervention (Table 2).

The COPM after HAL intervention increased in all patients compared with that before the intervention. COPM performance and satisfaction showed significant improvement after HAL (*p* = 0.043), 1 month after HAL (*p* = 0.043), and 3 months after HAL (*p* = 0.043) compared to before the HAL intervention. In patient 1, the satisfaction score was lower 1-month post-HAL; however, the score increased again 2–3 months after HAL intervention. In other cases, the COPM scores were maintained, or increased, 1–3 months after HAL intervention. Patients 2, 4, and 6 were difficult to measure for COPM because they had difficulty in verbalizing his condition and expressing their thoughts. Therefore, these details were requested from their mothers (Table 2).

Maximum isometric knee extension strength showed significant improvement 1 month after HAL intervention (*p* = 0.0496) and 2 months after HAL intervention (*p* = 0.027) compared to preoperatively. Furthermore, compared to before HAL intervention, there was a significant improvement at 1 month after HAL intervention (*p* = 0.046) and at 3 months after HAL intervention (*p* = 0.046). For lower limb muscle strength, follow-up evaluation of the HAL intervention was not performed in patient 3. In patient 4, it was difficult to measure the maximum isometric knee extension torque because of be performed the test movements could not be understood (Table 2).

In patient 1, gait speed, step length, and cadence decreased after the Achilles tendon lengthening surgeries. After the HAL intervention, walking speed, step length, and cadence increased compared with values before the intervention. The SWS and step length on SWS improved to preoperative values at 1 month after HAL intervention. Although cadence was abnormally high, with a preoperative value of 140 steps/min, it decreased to 130 steps/min 1 month following HAL intervention. Although cadence was low in the MWS post-surgery, it improved to the preoperative value after the HAL intervention. The step length in MWS improved compared with the preoperative value before HAL intervention, and it continued to increase 3 months after HAL. In patients 2 and 3, the SWS before HAL intervention was not reduced compared with the preoperative value. The cadence before HAL intervention drastically increased (Table 2).

Regarding walking endurance, patients 1 and 2 showed a decrease in 6MD in the postoperative period. This improved after HAL intervention and was restored to the preoperative value 2 months post-HAL intervention (Table 2).

The mean gait time and distance in each session were 17.6 (range, 14.8–20.7) min and 376 (range, 147–560) m, respectively. The Borg scale score was 11.9 (range, 11.0–13.0) for the degree of fatigue during training. The range of motion of the affected joints pre- and post-gait training using HAL improved in five cases compared with the preoperative range of motion. One patient showed a decrease in the range of motion of the affected joints post-gait training using HAL, although a follow-up assessment 1 month later using HAL showed improvement in the affected joints (Table 3).

**Figure 3:**
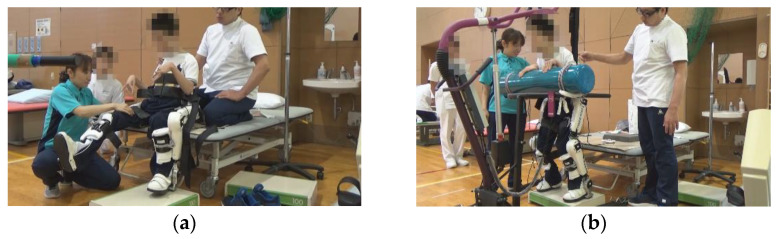
Exercises with the Hybrid Assistive Limb^®^ (HAL) device. (**a**) Knee flexion and extension exercise in a sitting position with the Hybrid Assistive Limb^®^ (HAL). (**b**) Squat exercise in a standing position with HAL.

In patient 5, a patient with quadriplegia, pain occurred during knee flexion and extension due to postoperative immobilization in a cast. Thus, we started with knee flexion and extension in a sitting position with pain control, then in the squat position, and finally in a standing position (Figure 3).

## 4. Discussion

All participants completed the protocol of 12 sessions gait training intervention with HAL In addition, no adverse events were observed after gait training with HAL. Therefore, gait training with HAL is a safe and feasible intervention for patients who undergo soft tissue surgeries. In this patient, there was improvement in GMFM, COPM, and lower limb muscle strength after HAL intervention. It may be useful to integrate training using HAL as part of a postoperative rehabilitation program for CP.

The primary endpoint, GMFM, showed improvement at immediately after, 1 month and 3 months after HAL intervention compared to before HAL intervention. In addition, improvement was shown 3 months after HAL intervention compared to preoperatively. These results indicate that HAL intervention after surgery improves gross motor function compared to preoperatively, not only immediately after HAL intervention, but also at 3 months after HAL intervention. In this study, postoperative rehabilitation during the HAL training period was not HAL alone, but a combination of usual rehabilitation and HAL training. Therefore, the impact of postoperative functional improvement in this case could be attributed to the improvement due to HAL intervention, the improvement due to surgery, and the improvement due to usual postoperative rehabilitation, respectively. On the natural course of the postoperative period, in CP patients who underwent Achilles’ tendon lengthening, GMFM decreased at 6 and 12 months postoperatively, and it took 24 months for GMFM to recover [15]. Regarding usual postoperative rehabilitation, Vasileios et al. reported that postoperative physical therapy increased GMFM at 9 months [16]. On the other hand, one year after surgery, GMFM had recovered to preoperative values, but surgery did not improve GMFM [17]. Seniorou et al. reported a significant decrease in GMFM six months after surgery despite usual physical therapy, and six weeks of strength training six months after surgery improved GMFM, but GMFM decreased again one year after surgery [18]. However, Seniorou et al. included a case in which an osteotomy was performed, which may have influenced in a difference in the surgical method [18]. In this study, GMFM recovered in a shorter recovery period compared to the typical surgical course [15,16,17,18]. Therefore, incorporating HAL intervention as part of postoperative rehabilitation may help for faster recovery.

In terms of subjective evaluation, performance and satisfaction of the COPM markedly increased when comparing conditions before and after the HAL intervention, despite the patients not undergoing a tendon lengthening procedure. During usual physiotherapy post-operation, there are several cases wherein the pain occurs during joint motion in the muscles that were operated on due to contracture. In our cases, the improvement in the subjective evaluation may have occurred because the patients were able to perform repeated lower limb movements without pain by adjusting the assistance of HAL.

Regarding knee extension strength, even in cases where maximum isometric knee extension strength decreased postoperatively, the preoperative value in all patients was restored within 3 months after the intervention. Even with muscle strength training post-surgery, knee extension strength was lower at 6 months postoperatively, and recovery took 1 year [19]. In the present case, the effectiveness of HAL intervention in postoperative training was demonstrated, as it allowed recovery to the preoperative value 6 months postoperatively.

In this case, the patient had limited ambulatory CP, and the data obtained regarding walking ability and walking endurance were negligible. 10MWT was measured only in patients with GMFCS I–III who were able to walk 10 m without assistance, because this study included patients with severe CP of GMFCS level III–IV. 6MWT was measured in two patients with GMFCS level I who were able to walk long distances. GMFCS level III who was able to walk short distances, although he was a tendency to obese and had difficulty walking long distances. Previous reports confirmed improvement in joint angle and stride length during gait 1-year post-orthopedic surgery, whereas improvement in gait speed required 2 years [20]. In this case, by using HAL as a means of postoperative rehabilitation, the improvement in walking ability was recognized early, at 4–5 months postoperatively. Hence, HAL intervention after orthopedic surgery may accelerate the recovery of walking ability. Regarding walking endurance, 6MD of two hemiplegia CP was restored to the preoperative value 4–5 months postoperatively. The PCI decreased to preoperative levels 4 months post-surgery in one of the two patients in whom the PCI was measured; however, it increased from preoperative levels in one patient. There are few reports on the course of walking endurance after orthopedic surgery, although it has been reported that it takes up to 2 years to improve oxygen consumption relative to energy cost during walking after orthopedic surgery [21]. Although the data in this study is limited, with only two participants able to measure 6MD, the value of 6MD 5 months postoperatively exceeded that of preoperative 6MD. Using HAL assist for long-distance walking in the early postoperative period may improve postoperative walking endurance.

Gait training with HAL was performed for an average of 1.5 months post-surgery. When Lokomat is used as the walking practice in the postoperative period, intervention at 1.5–2 months post-operation has been recommended; thus, the HAL intervention starting time in the postoperative course of this study may be appropriate [22].

Cast fixation is typically performed for postoperative pain reduction and wound stabilization, and active movement of the joint is initiated after the pain is controlled; generally, upon standing or walking during rehabilitation. Wound healing generally takes approximately 2–4 weeks, and gait training can be delayed while allowing time for sufficient wound healing. However, prolonged periods of immobilization in patients with intrinsic muscle weakness can worsen muscle weakness itself [23]. Early gait training may be possible using HAL. Nevertheless, HAL was reported to be difficult to wear during gait training in one case owing to lower limb deformation [7]. In such cases, it is difficult to repeat the correct direction of movement using HAL, and the feedback effect through HAL may not be received effectively. Therefore, we recommend surgical intervention before using HAL for gait training. In two patients with GMFCS level IV, gait training with conventional post-surgery physical therapy was not initiated because of pain in the surgical site, although the HAL intervention enabled them to perform gait training for the first time after surgery. In GMFCS level IV CP, major assistance is needed for standing and walking. Our results suggest that HAL intervention while adjusting HAL assistance to prevent pain, enables standing and walking sooner after surgery; thus, patients may be able to learn normal joint movements.

In all patients, the cause of range of motion improvement in the affected joints was surgery. Despite postoperative pain and muscle weakness, joint contractures and deformities did not recur. As we have shown, this may be the result of achieving a normal gait pattern and muscle strengthening through HAL. In a rehabilitation study using HAL after total knee arthroplasty, normal gait pattern, improved muscle strength, and less postoperative pain were observed [24]. This suggests that HAL helps prevent muscle contraction associated with pain and improves the range of motion expansion and walking ability. Similarly, in this study, improvement in walking ability after HAL training can be attributed to HAL’s gait learning effect [5,6,7]. Future studies should determine cases where HAL can be used by assessing a greater number of patients and recording periodic changes.

In a report on Lokomat training recommendations, when the robot was used primarily to improve walking ability, patients with severe intellectual disabilities did not show sufficient improvement, and adaptation to robot training was reported to be low [22]. In patients with GMFCS level IV or severe intellectual disability, measurable outcomes were limited, and the improvement effect using HAL was not sufficiently recognized. However, the GMFM, PEDI, and COPM showed improvement after HAL intervention, even in patients with severe CP. Therefore, we suggest that the HAL intervention effect became clearer by including more relevant measurable outcomes in patients with severe intellectual disability and/or motor function.

This study had some limitations. First, surgery was performed in a small number of cases, and no matched controls were included. Moreover, data quantity of results was substantially lacking for walking ability and endurance. On the other hand, it is difficult to collect many patients presenting similar walking abilities in conjunction by orthopedic surgery and HAL intervention because of large variations in medical conditions and motor functions in CP patients. Second, the study had a short follow-up duration; therefore, this study provided no data on whether these improvements were comparable to 1 or 2 years of conventional rehabilitation. Third, only patients who underwent tendon and muscle lengthening were evaluated; therefore, those who had undergone other orthopedic surgeries, such as osteotomy, were not included. Future studies should enroll more cases, comparing the HAL intervention group with the conventional rehabilitation group after surgery. Moreover, we need to evaluate the effectiveness of HAL training after different types of orthopedic surgeries.

## 5. Conclusions

In conclusion, we assessed the short-term outcomes of a rehabilitation program with HAL after multi-level soft tissue lengthening in young patients with CP. Improvements in motor function, walking ability, and subjective assessment are likely due to this rehabilitation program with HAL.

## Figures and Tables

**Table 1:** Patient characteristics and surgical details.

Patient No.	1	2	3	4	5	6
Sex	Male	Female	Male	Female	Male	Male
Age (years)	11	13	17	16	16	24
Height (cm)	131.6	151.2	163.6	144.5	152	165
Weight (kg)	33.8	34.5	79.7	44.7	34.6	45.1
GMFCS level ^1^	I	II	III	IV	IV	IV
Type of CP ^2^	R ^3^ Hemiplegia	R ^3^ Hemiplegia	Diplegia	Diplegia	Quadriplegia	Quadriplegia
Reason for surgery	Equinus	Equinovarus	Equinus	Equinovarus	Crouch	Crouch
Surgery	ATL ^4^	ATL ^4^, TPTL ^5^	ATL ^4^	ATL ^4^, TPTL ^5^	HL ^6^	HL ^6^
Period of casting (days)	14	35	15	21	28	42
Period of starting HAL ^7^ training from surgery (days)	36	55	40	40	62	64
Period of HAL ^7^ training (days)	20	23	19	29	23	27
Period of hospital stay (days)	58	87	78	82	114	161

^1^ GMFCS: Gross Motor Function Classification System; ^2^ CP: Cerebral palsy; ^3^ R: Right; ^4^ ATL: Achilles’ tendon lengthening; ^5^ TPTL: Tibialis posterior tendon lengthening; ^6^ HL: Hamstring lengthening; ^7^ HAL: hybrid assistive limb.

**Table 2:** Motor function outcome measures.

Outcome		Measured Period	Average	Median	Standard Deviation	Wilcoxon Signed-Rank Tests *p*	
						Comparison with Ope Pre	Comparison with Pre HAL
**GMFM ^1^ total**	% (0–100)	Ope pre	66.37	68.75	24.51	-	-
		Pre-HAL	59.77	62.65	30.70	0.310	-
		Post-HAL	65.70	65.30	26.84	0.753	0.027 *
		Post-HAL after 1 month	64.00	48.10	25.55	0.345	0.043 *
		Post-HAL after 2 months	68.55	67.60	26.71	0.068	0.068
		Post-HAL after 3 months	66.20	56.80	24.33	0.043 *	0.042 *
**GMFM ^1^** **Dimension C** **(crawl and kneel)**	Score (0–42)	Ope pre	26.33	31.50	13.89	-	-
		Pre-HAL	21.00	20.00	16.77	0.416	-
		Post-HAL	23.67	21.00	14.46	0.715	0.109
		Post-HAL after 1 month	22.80	19.00	12.07	1.000	0.109
		Post-HAL after 2 months	23.75	21.00	13.33	0.180	0.180
		Post-HAL after 3 months	24.60	29.00	12.34	0.655	0.109
**GMFM ^1^** **Dimension D** **(stand)**	Score (0–39)	Ope pre	18.83	17.00	13.89	-	-
		Pre-HAL	17.50	16.50	15.31	0.599	-
		Post-HAL	19.50	19.00	15.75	0.686	0.026 *
		Post-HAL after 1 month	18.40	7.00	15.28	0.785	0.043 *
		Post-HAL after 2 months	22.25	21.50	15.29	0.102	0.068
		Post-HAL after 3 months	20.00	10.00	14.35	0.141	0.043 *
**GMFM ^1^** **Dimension E** **(walk, run and jump)**	Score (0–72)	Ope pre	29.67	24.50	28.65	-	-
		Pre-HAL	28.50	22.00	29.24	0.273	-
		Post-HAL	31.50	26.50	29.70	0.246	0.042 *
		Post-HAL after 1 month	29.00	6.00	31.15	0.197	0.066
		Post-HAL after 2 months	35.50	35.00	31.60	0.066	0.066
		Post-HAL after 3 months	30.20	8.00	30.15	0.043 *	0.043 *
**PEDI ^2^**	Score (0–197)	Ope pre	125.40	123.00	46.18	-	-
		Pre-HAL	121.40	123.00	50.17	0.180	-
		Post-HAL	121.60	123.00	50.35	0.317	0.317
		Post-HAL after 1 month	126.20	123.00	50.13	0.593	0.066
		Post-HAL after 2 months	139.50	145.00	47.51	0.180	0.102
		Post-HAL after 3 months	127.80	123.00	48.47	0.180	0.068
**COPM ^3^ performance**	Score (1–10)	Pre-HAL	2.84	2.40	1.08	-	-
		Post-HAL	5.96	5.40	1.37	-	0.043 *
		Post-HAL after 1 month	6.80	6.20	1.29	-	0.043 *
		Post-HAL after 2 months	7.70	7.90	1.64	-	0.068
		Post-HAL after 3 months	8.40	9.20	1.72	-	0.043 *
**COPM ^3^ satisfaction**	Score (1–10)	Pre-HAL	2.16	1.40	1.24	-	-
		Post-HAL	5.96	5.80	2.05	-	0.043 *
		Post-HAL after 1 month	6.56	6.60	1.25	-	0.043 *
		Post-HAL after 2 months	7.90	8.10	1.72	-	0.068
		Post-HAL after 3 months	8.04	8.60	1.58	-	0.043 *
**Maximum isometric knee extension strength**	Nm	Ope pre	36.10	39.50	16.64	-	-
		Pre-HAL	36.38	38.00	12.60	0.310	-
		Post-HAL	37.30	36.50	20.31	0.878	0.069
		Post-HAL after 1 month	40.00	35.00	17.91	0.0496 *	0.046 *
		Post-HAL after 2 months	42.33	34.00	20.50	0.027 *	0.109
		Post-HAL after 3 months	40.75	41.00	14.67	0.092	0.046 *
**10MWT ^4^ (SWS ^5^)**	Gait speed (m/min)	Ope pre	54.27	42.00	20.24	-	-
		Pre-HAL	55.93	57.60	7.52	-	-
		Post-HAL	64.30	64.70	5.97	-	-
		Post-HAL after 1 month	74.10	74.10	9.90	-	-
		Post-HAL after 2 months	69.07	69.80	3.71	-	-
		Post-HAL after 3 months	84.35	84.35	32.05	-	-
	Step length (cm)	Ope pre	48.33	46.00	7.93	-	-
		Pre-HAL	45.00	45.00	6.53	-	-
		Post-HAL	55.00	56.00	6.98	-	-
		Post-HAL after 1 month	59.50	59.50	3.50	-	-
		Post-HAL after 2 months	56.00	59.00	4.24	-	-
		Post-HAL after 3 months	59.50	59.50	3.50	-	-
	Cadence (step/min)	Ope pre	108.83	95.10	22.60	-	-
		Pre-HAL	124.20	124.30	2.16	-	-
		Post-HAL	119.10	125.00	11.22	-	-
		Post-HAL after 1 month	125.10	125.10	9.50	-	-
		Post-HAL after 2 months	123.77	124.10	4.01	-	-
		Post-HAL after 3 months	112.50	112.50	18.30	-	-
**10MWT ^4^ (MWS ^6^)**	Gait speed (m/min)	Ope pre	81.60	81.60	14.40	-	-
		Pre-HAL	88.10	88.10	3.50	-	-
		Post-HAL	95.75	95.75	14.05	-	-
		Post-HAL after 1 month	87.35	87.35	17.05	-	-
		Post-HAL after 2 months	103.45	103.45	24.35	-	-
		Post-HAL after 3 months	95.85	95.85	20.55	-	-
	Step length (cm)	Ope pre	59.50	59.50	3.50	-	-
		Pre-HAL	61.50	61.50	5.50	-	-
		Post-HAL	70.00	70.00	7.00	-	-
		Post-HAL after 1 month	70.00	70.00	7.00	-	-
		Post-HAL after 2 months	73.00	73.00	10.00	-	-
		Post-HAL after 3 months	71.00	71.00	12.00	-	-
	Cadence (step/min)	Ope pre	131.00	131.00	10.00	-	-
		Pre-HAL	146.00	146.00	19.00	-	-
		Post-HAL	137.00	137.00	6.00	-	-
		Post-HAL after 1 month	124.50	124.50	11.50	-	-
		Post-HAL after 2 months	140.00	140.00	14.00	-	-
		Post-HAL after 3 months	134.00	134.00	6.00	-	-
**6MWT (m) ^7^**		Ope pre	435.00	435.00	18.00	-	-
		Pre-HAL	363.50	363.50	9.50	-	-
		Post-HAL	415.50	415.50	25.50	-	
		Post-HAL after 1 month	399.00	399.00	1.00	-	-
		Post-HAL after 2 months	489.00	489.00	39.00	-	-
		Post-HAL after 3 months	468.50	468.50	14.50	-	-
**PCI (beat/m) ^8^**		Ope pre	0.45	0.45	0.05	-	-
		Pre-HAL	0.50	0.50	0.10	-	-
		Post-HAL	0.65	0.65	0.05	-	-
		Post-HAL after 1 month	0.35	0.35	0.15	-	-
		Post-HAL after 2 months	0.55	0.55	0.05	-	-
		Post-HAL after 3 months	0.50	0.50	0.10	-	-

^1^ GMFM: Gross Motor Function Measure; ^2^ PEDI: Pediatric Evaluation of Disability Inventory; ^3^ COPM: Canadian Occupational Performance Measure, ^4^ 10MWT: 10 m walking test; ^5^ SWS: self-selected walking speed; ^6^ MWS: maximum walking speed; ^7^ 6MD: 6 min walking distance; ^8^ PCI: physiological cost index; * *p*  <  0.05.

**Table 3:** Clinical outcomes.

Patient No.	Measured Period	1	2	3	4	5	6
**Surgery**		ATL ^2^ and/or TPTL ^3^				HL ^4^	
**ROM ^1^** **(right/left) (°)**		Ankle dorsiflexion				Knee extension	
	Ope pre	−5/15	−20/10	−20/−30	−10/−10	−40/−40	−60/−70
	Pre-HAL	20/5	0/10	5/5	10/0	−20/−10	−35/−35
	Post-HAL	20/10	5/10	10/20	−10/−10	−10/−10	−40/−40
	Follow-up				10/10	-	-

^1^ ROM: Range of motion; ^2^ ATL: Achilles’ tendon lengthening; ^3^ TPTL: Tibialis posterior tendon; ^4^ HL: Hamstring lengthening.

## Data Availability

Not applicable.
